# Comparative Effects of Copper Glycinate and Potassium Diformate on Immunity and Gut Microbiota of Pigs—Potential Analysis of Potassium Diformate as a Copper Additive Substitute

**DOI:** 10.3390/ani16121889

**Published:** 2026-06-18

**Authors:** Xueyuan Jiang, Hulong Lei, Yuan Mei, Peng Jia, Wen Yao, Dong Xia, Naisheng Lu

**Affiliations:** 1 Key Laboratory of Livestock and Poultry Resources (Pig) Evaluation and Utilization of Ministry of Agriculture and Rural Affairs, Shanghai Engineering Research Center of Breeding Pig, Institute of Animal Science and Veterinary Medicine, Shanghai Academy of Agricultural Sciences, Shanghai 201106, China; jiangxueyuan@saas.sh.cn (X.J.); leihulong@saas.sh.cn (H.L.); mymymzl@126.com (Y.M.); jiapeng@saas.sh.cn (P.J.); 2 College of Animal Science &Technology, Nanjing Agricultural University, Nanjing 210095, China; yaowen67jp@njau.edu.cn

**Keywords:** copper, diet, feed additive, immunity, microbiota, potassium diformate

## Abstract

Copper supplements are prominent antibiotic alternatives in livestock production, but their excessive use poses environmental risks, necessitating safer substitutes. Potassium diformate (KDF), an organic acid salt, has shown potential as a feed additive, yet its effectiveness in copper-unsupplemented diets remains unclear. This study compared the effects of copper glycinate (Cu_Gly) and KDF on weaned piglets fed a copper-unsupplemented basal diet. Forty-five Meishan piglets were allocated to a control group (basal diet), a Cu_Gly group (basal diet + 60 mg/kg Cu_Gly), or a KDF group (basal diet + 10 g/kg KDF). The KDF group exhibited a lower feed conversion ratio (FCR) than the control group. Although no significant differences in average daily gain were observed, KDF supplementation significantly enhanced immune function by increasing plasma IgG and decreasing IgM, IL-6, and IL-1 levels compared to the control. 16S rRNA sequencing analysis revealed that the Control group was enriched with pathogenic genera (*Staphylococcus*, *Escherichia-Shigella*, *Brevibacterium*) in the ileum and cecum. Cu_Gly supplementation enriched *Bacillus*, *Longispora*, and *Terrisporobacterin* the ileum, while KDF increased the abundance of beneficial taxa (*TM7*, *Nesterenkoniain* the ileum; *Lachnospiraceae_UCG-007* in the cecum). Correlation analysis showed that the abundances of *Escherichia-Shigella* and *Staphylococcus* were negatively correlated with IgG but positively correlated with IgM and IL-6. Cecal *Lachnospiraceae_UCG-007* (enriched in the KDF group) was negatively correlated with IL-1. In summary, KDF improved feed efficiency and demonstrates superior immunomodulatory effects compared to Cu_Gly. The enrichment of bacteria linked to metal resistance in the Cu_Gly group suggested a potential drawback not associated with KDF. Therefore, KDF represented a viable and sustainable alternative to copper supplements, offering a combination of improved gut health, enhanced immunity, and a reduced environmental risk profile.

## Simple Summary

While copper supplements are common antibiotic alternatives in livestock, their environmental impact drives the search for safer options. This study evaluated potassium diformate (KDF) as a potential substitute for copper glycinate (Cu_Gly) in weaned piglet diets without added copper. Both additives reduced gut pathogenic bacteria. However, Cu_Gly carried the risk of increasing the abundance of copper-resistant bacteria. In contrast, KDF not only improved feed efficiency but also enhanced the immune response by modulating gut microbiota, increasing IgG and decreasing pro-inflammatory markers. Therefore, KDF presented a safer and more sustainable alternative, promoting gut health and immunity without the associated risks of copper resistance.

## 1. Introduction

The mammalian gastrointestinal tract harbors trillions of commensal microorganisms that exert a profound influence on host physiological homeostasis, including digestion, metabolism, and immunity [1]. The gut microbial communities assemble shortly after birth and build up a milk-oriented microbiota in suckling piglets. However, the weaning transition disrupts the stable neonatal microbiota that is adapted to milk. The concurrent changes in maternal separation and introduction of solid diet lead to a rapid restructuring of the microbial assemblage, which can result in dysbiosis [2,3]. Consequently, weaned piglets are more susceptible to pathogens and are prone to frequent episodes of diarrhea [4]. High-dose copper salts (100–250 mg/kg) have long been used as antibiotic alternatives in weaned piglet diets due to their strong antibacterial activity [5,6,7]. Højberg et al. (2005) reported that 175 mg/kg copper from copper sulfate inhibited intestinal Escherichia coli and improved the growth performance of newly weaned piglets [8]. Similarly, Wang et al. (2012) found that 100 mg/kg copper from copper-loaded chitosan nanoparticles reduced *Escherichia coli* counts while increasing beneficial *Lactobacillus* and *Bifidobacterium* abundances in the intestinal tract [9]. However, the low bioavailability of copper salts results in high fecal copper excretion, causing soil accumulation, water contamination, and potential food chain risks [10,11]. These environmental concerns have driven the search for effective and eco-friendly alternatives to high-copper supplements.

Potassium diformate (K-diformate, KDF), a unique conjugated acid salt (HCOOH HCOOK), exhibits significant antimicrobial properties and has been used as a feed additive for pigs [12,13,14]. In vitro trials showed that KDF directly inhibited the proliferation of coliforms and lactic acid bacteria [15]. In vivo trials demonstrated that KDF supplementation reduced the abundance of total anaerobes, coliforms, and *Salmonella* in the gastrointestinal tract of piglets [16,17,18], while decreasing diarrhea incidence and improving growth performance [19,20]. Notably, most previous studies on KDF were conducted in diets supplemented with copper, leaving a knowledge gap regarding its effects in copper-unsupplemented diets.

Organic copper sources (e.g., copper glycinate) have higher bioavailability than inorganic forms [21]. Research indicated that 50–80 mg/kg organic copper could achieve similar growth-promoting effects to 150–250 mg/kg inorganic copper [11,22,23]. Based on this, the present study used 60 mg/kg Cu from copper glycinate (Cu_Gly) as a reference and compared its effects with 10 g/kg KDF on growth performance, immune function, and gut microbiota in weaned piglets fed copper-unsupplemented basal diets. The goal was to evaluate KDF’s potential as a sustainable alternative to copper additives.

## 2. Materials and Methods

### 2.1. Ethics Statement

This experiment was approved by the Institutional Animal Care and Use Committee (IACUC) of Shanghai Academy of Agricultural Sciences (Grant number: SAASPZ0520011). All animal care and experimental protocols were conducted according to the guidelines of the Laboratory Animal-Guideline for Ethical Review of Animal Welfare (GB/T 35892-2018) set by the Standardization Administration of China [24].

### 2.2. Animals

The experimental pig houses consisted of two rows of pens with 10 pens in each row. We used 15 pens in sequence. A total of 45 Meishan female piglets weaned at 45 days were allocated randomly into three groups paired in litter and body weight (5 replicate pens/group, and 3 piglets/pen). Piglets in each group received either a basal diet without any Cu additive (control group), or basal diet supplemented with 60 mg/kg Cu_Gly (Cu_Gly group), or a basal diet supplemented with 10 g/kg KDF ((Formi, KDF ≥ 95.6%, and silicon dioxide ≤ 1.5%, Addcon (Dalian) Environmental Products, Dalian, China) (KDF group). Basal diets were formulated based on local breeds’ nutrient requirements and physiological needs (Table 1). The piglets had ad libitum access to diets and water for seven weeks. The body weight and feed consumption were recorded at the beginning and end of the trial. The average daily body weight gain (ADG) and average daily feed intake (ADFI) were calculated as the total body weight gain and total feed intake divided by the number of experimental days, respectively. The feed conversion ratio (FCR) was determined as the ratio of total feed intake to total body weight gain.

### 2.3. Sample Collection

At the end of the experiment, we selected five piglets whose body weight was closest to the median within that pen for sampling from each treatment and sacrificed via exsanguination following euthanasia. Subsequently, blood was collected with EDTA-coated tubes, and plasma was obtained by centrifugation at 3000 rpm for 20 min and stored at −20 °C until cytokine and immunoglobulin analysis. The digesta of the ileum and cecum were collected into sterile plastic tubes and stored at −80 °C until microbial DNA extraction. The liver and spleen were dissected, blotted dry, and weighed to calculate relative organ weights (g/kg BW).

### 2.4. Determination of Immunoglobulin and Cytokines Contents in Plasma

The contents of immunoglobulin (IgA, IgG, IgM) in plasma were measured using the commercial ELISA kits (Nanjing Jiancheng Bioengineering Institute, Nanjing, China). Plasma cytokines (IL-1, IL-2, IL-4, IL-6, IL-10, TNF-α, IFN-γ) were detected using the commercial ELISA kits of Beijing Solarbio Science & Technology Co., Ltd. (Beijing, China). All samples were assayed in triplicate.

### 2.5. 16S rRNA Gene Sequencing and Microbiota Analysis

Total genome DNA from ileum and cecum digesta was extracted using commercial stool DNA extraction kits (Solarbio Science & Technology Co., Ltd., Beijing, China) following the manufacturer’s instructions. The extracted genomic DNA concentration and purity were determined using a NanoDrop 1000 spectrophotometer (Thermo Fisher Scientific, Wilmington, DE, USA). DNA amplification was performed by using a barcode-tagged primer set for pyrosequencing of the bacterial 16S rRNA gene. This primer set targeted the V4 and V5 hypervariable regions of the 16S rRNA genes using the 515F (5′-GTGCCAGCMGCCGCGG-3′) and 907R (5′- CCGTCAATTCMTTTRAGTTT-3′). The PCR was performed using 4 μL 5 × FastPfu Buffer, 2 μL dNTPs (2.5 mM), 0.4 μL FastPfu Polymerase, 0.8 μL of each primer (5 μM), and 10 ng of fecal DNA as the template. The amplification program consisted of an initial denaturation at 95 °C for 5 min, 27 cycles at 95 °C for 30 s, 55 °C for 30 s, 72 °C for 45 s, and a final extension at 72 °C for 10 min. The amplification products from each sample were evaluated by electrophoresis in 2% agarose gel and purified using the QIAquick PCR purification kit (Qiagen, Valencia, CA, USA). The products were quantified using QuantiFluorTM-ST fluorescent quantitation system (Promega, Madison, WI, USA) and mixed in equivalent proportions. Sequencing was performed using Illumina Miseq PE250 (Illumina, Inc., San Diego, CA, USA) according to the manufacturer’s instructions. A quality control of sequences was conducted, and only high-quality sequences were used for subsequent analyses. The operational taxonomic units (OTU) picking with 97% similarity cut-off was compiled with Qiime using default parameters. Taxonomic classification was performed based on the OTU database. Alpha diversity index (Shannon) and β-diversity (Bray–Curtis) analyses were calculated using the phyloseq package (1.46) in R version (4.2.3). Principal coordinates analysis (PCoA) based on the Bray–Curtis distance matrix was performed with the Mothur program (http://www.mothur.org, accessed on 23 October 2024). The linear discriminant analysis effect size (LEfSe) analysis was conducted by LEfSe software (2.2.0).

### 2.6. Statistical Analysis

Data normality and variance homogeneity were tested using the Shapiro–Wilk and Levene tests, respectively. Growth performance, organ weights, and plasma immune indices were analyzed by one-way ANOVA with Tukey’s multiple comparison test using SPSS 17.0 (IBM, Armonk, NY, USA). Microbial alpha diversity indices and relative abundances were analyzed using the Kruskal–Wallis test. The data were presented as the means ± standard error (SE). Pearson correlation analysis was performed to assess relationships between microbial genera and immune indices. Statistical significance was set at *p* < 0.05, and 0.05 ≤ *p* < 0.10 was considered a tendency.

## 3. Results

### 3.1. Growth Performance and Relative Organ Weight

As shown in Table 2, there was no significant difference in the body weight, ADG and ADFI among the three groups. However, the KDF group had a significantly lower FCR than the Control group (*p* < 0.05), with no difference between Cu_Gly and KDF groups (*p* > 0.05).

At the end of the experiment, the Cu_Gly group had a higher relative liver weight than the Control group (*p* < 0.05), while the KDF group showed no significant difference compared to the other two groups (*p* > 0.05). Both Cu_Gly and KDF groups exhibited lower relative spleen weights than the Control group (*p* < 0.05), with no difference between the two treatment groups (*p* > 0.05).

### 3.2. Immunoglobulin and Cytokines

As shown in Table 3, the KDF treatment had significantly higher plasma IgG content and lower plasma content of IgM and IL-6 than that of the control and Cu_Gly group (*p* < 0.05). Additionally, KDF supplementation reduced plasma IL-1 levels compared to the Control group (*p* < 0.05). No significant differences were observed in IgA, IL-2, IL-4, IL-10, TNF-α, or IFN-γ concentrations among the three groups (*p* > 0.05). The Control and Cu_Gly groups showed no differences in any detected immune indices (*p* > 0.05).

### 3.3. Ileum and Cecum Microbial Communities

The characteristics of ileum and cecum microbial communities were analyzed using 16S sequencing (Table 4). The α-diversity analysis revealed distinct effects of treatments on ileal but not cecal microbiota. The Cu_Gly group had higher ileal OTU, Chao, Shannon, and Simpson indices than the Control group (*p* < 0.05). The KDF group showed a tendency toward increased ileal Shannon (*p* = 0.056) and Simpson (*p* = 0.054) indices compared to the Control group, with no significant differences from the Cu_Gly group (*p* > 0.05). No differences in cecal alpha diversity indices were observed among groups (*p* > 0.05).

**Figure 1:**
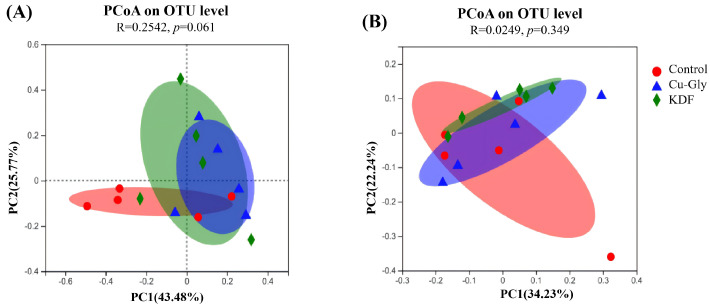
PCoA of the weighted UniFrac distances for control, Cu_Gly group and KDF groups (n = 5). (**A**) Ileum content and (**B**) cecum content. Control, basal diet. Cu_Gly, basal diet + 60 mg/kg Cu-Glycine. KDF, basal diet + 10 g/kg potassium diformate.

**Figure 2:**
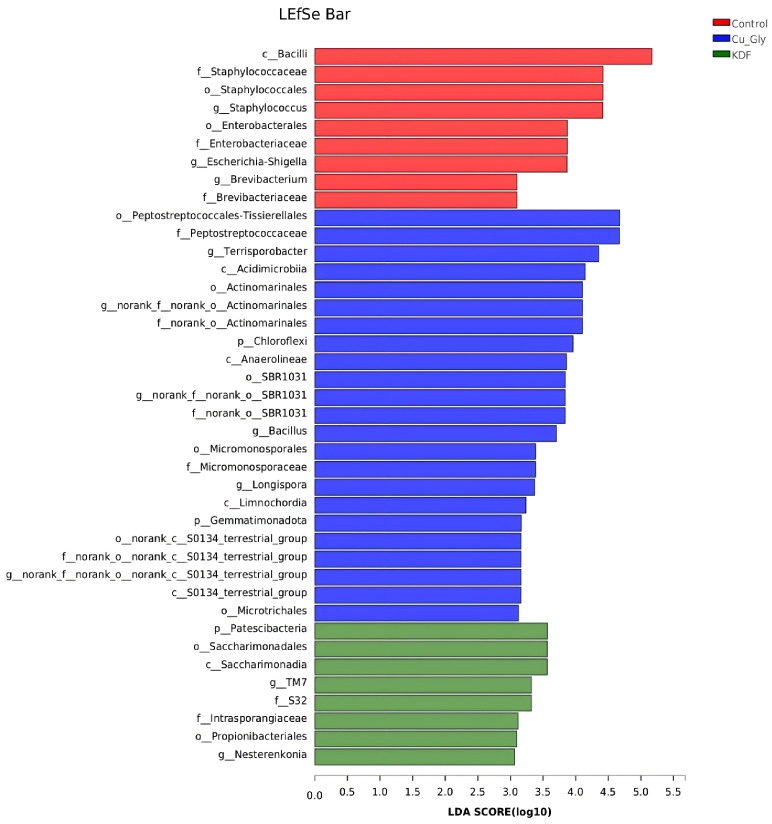
LEfSe analysis of ileum bacteria filtered out the biomarkers of the microbial community for control, Cu_Gly group and KDF groups (n = 5). LDA SCORE (log10) ≥ 3.0.

**Figure 3:**
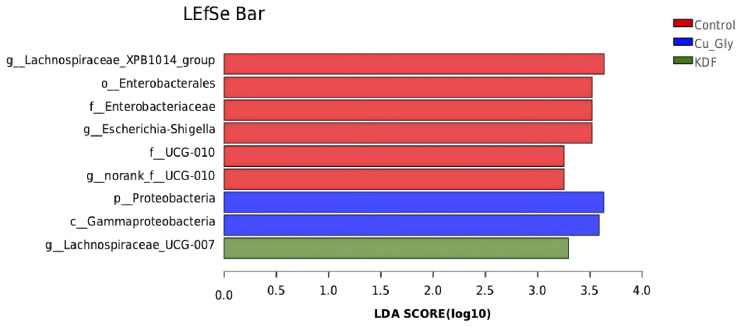
LEfSe analysis of cecum bacteria filtered out the biomarkers of the microbial community for control, Cu_Gly group and KDF groups (n = 5). LAD SCORE (log10) ≥ 3.0.

PCoA based on weighted UniFrac distances showed no significant separation of ileal or cecal microbial communities among groups (*p* > 0.05, Figure 1). LEfSe analysis identified group-specific microbial biomarkers (Figure 2 and Figure 3). In the ileum (Figure 2), the Control group enriched with *bacilli* class, *Staphylococcales* and *Enterobacterales* order, *Staphylococcaceae* and *Enterobacteriaceae* family, *Staphylococcus* and *Escherichia-Shigella* and *Brevibacterium* genera. The Cu_Gly group enriched with two phyla (*Chloroflexi*, *Gemmatimonadota*), four bacterial classes (*Acidimicrobiia*, *Anaerolineae*, *Limnochordia*, *S0134_terrestrial_group*), six bacterial orders (*Peptostreptococcales-Tissierellales*, *Actinomarinales*, *SBR1031*, *Micromonosporales*, *norank_c_S0134_terrestrial_group*, *Microtrichales*), six bacterial family (*Brevibacteriaceae*, *Peptostreptococcaceae*, *norank_o_Actinomarinales*, *norank_o_SBR1031*, *Micromonosporaceae*, *norank_o_norank_c_S0134_terrestrial_group*), and six genera (*Terrisporobacter*, *norank_f_norank_o_Actinomarinales*, *norank_f_norank_o_SBR1031*, *Bacillus*, *Longispora*, *norank_f_norank_o_norank_c_S0134_terrestrial_group*). The KDF group enriched with *Saccharimonadia* class, *Saccharimonadales* and *Propionibacteriales* order, *S32* and *Intrasporangiaceae* family, *TM7* and *Nesterenkonia* genera.

In the cecum content (Figure 3), the Control group enriched with *Proteobacteria* phylum, *Gammaproteobacteria* class, *Enterobacterales* order, *Enterobacteriaceae* and *UCG-010* family, *Lachnospiraceae_XPB1014_group*, *Escherichia-Shigella*, *norank_f_UCG-010*, and *Lachnospiraceae_UCG-007* genera. The Cu-Gly group is enriched with the *Proteobacteria* phylum and the *Gammaproteobacteria* class. In comparison, the KDF treatment enriched with the *Lachnospiraceae_UCG-007* genus.

### 3.4. Correlation Between Gut Microbiota and Immune Indices

**Figure 4:**
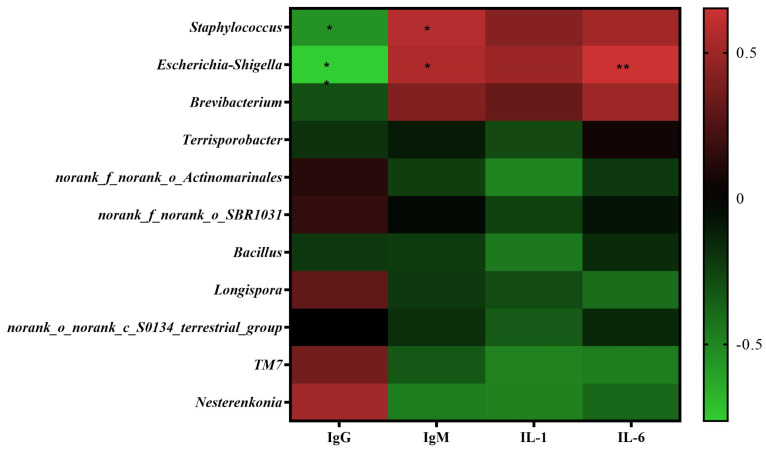
Pearson correlation analysis between the ileum bacteria at the genus level and the plasma content of immunoglobulin and cytokines was performed. Only significantly enriched bacteria and significant differential immunoglobulin and cytokines were analyzed. Control, basal diet. Cu_Gly, basal diet + 60 mg/kg Cu-Glycine. KDF, basal diet + 10 g/kg potassium diformate. * means *p* < 0.05, ** indicates *p* < 0.01, n = 5.

**Figure 5:**
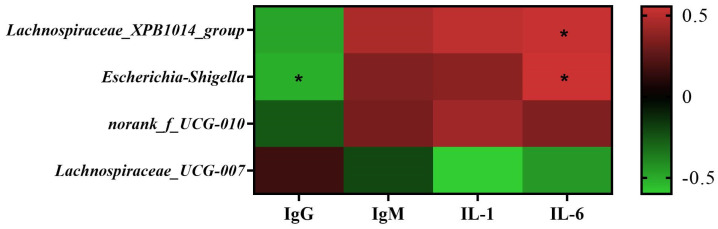
Pearson correlation analysis between the cecum bacteria at the genus level and the plasma content of immunoglobulin and cytokines. Only significantly enriched bacteria and significant differential immunoglobulin and cytokines were analyzed. Control, basal diet. Cu_Gly, basal diet + 60 mg/kg Cu-Glycine. KDF, basal diet + 10 g/kg potassium diformate. * means *p* < 0.05, n = 5.

Gut microbial genera and the plasma immune indices with significant differences among the three groups were subjected to Pearson’s correlation analysis (Figure 4 and Figure 5), the specific values are provided in Tables S1 and S2. In the ileum (Figure 4), the relative abundance of genus *Staphylococcus* and *Escherichia-Shigella* was negatively correlated with IgG (*p* < 0.05) but positively correlated with IgM (*p* < 0.05). Moreover, the abundance of the *Escherichia-Shigella* genus was positively correlated with IL-6 (*p* < 0.05). However, no significant correlations were observed between other differential genera (*Brevibacterium*, *Terrisporobacter*, *Actinomarinales*, *Bacillus*, *Longispora*, *TM7*, and *Nesterenkonia*) and immune indices (*p* > 0.05).

In the cecum (Figure 5), the abundance of the *Escherichia-Shigella* genus was negatively correlated with IgG (*p* <0.05) and positively correlated with IL-6 (*p* < 0.05). In addition, the *Lachnospiraceae_UCG-007* genus was negatively correlated with IL-1 content (*p* < 0.05), whereas the *Lachnospiraceae_XPB1014* genus was positively correlated with IL-6 (*p* < 0.05).

## 4. Discussion

The basal diet in this study contained 6.86 mg/kg intrinsic copper, which meets the nutritional requirement (5–6 mg/kg) for weaned piglets (NRC, 2012). However, plant-based copper often forms insoluble phytate complexes, reducing its bioavailability [25]. The inclusion of 500 FTU/kg phytase in the basal diet likely hydrolyzed these complexes, as the Control group showed similar ADG to the Cu_Gly and KDF groups. This was consistent with Suiryanrayna et al. (2015), who reported that phytase supplementation improves copper utilization and growth performance in piglets [26]. Organic acids could enhance phytase activity by lowering gut pH [25,27], but their interactive effects were varied by acid type and dosage. Valencia et al. (2002) found that phytase combined with acetic acid improves piglet growth [28], while Jongbloed et al. (2000) reported no synergistic effect between phytase and citric acid [29]. In the present study, 10 g/kg KDF did not affect ADG but reduced FCR, suggesting improved feed efficiency independent of phytase synergy. This differs from Février et al. (2001), who observed the negative effects of 18 g/kg KDF on phytase efficacy [30], highlighting the importance of KDF dosage optimization. Additionally, the production response in this trial was only reflected in an improved FCR for the KDF group compared to the control. This highlights its value as a supportive substance for newly weaned piglets. The economic and environmental sustainability of swine production depends on multiple factors beyond sheer growth rate. The significant enhancement of feed efficiency by KDF directly translates to lower feed costs per unit of weight gain.

The distinct effects of Cu_Gly and KDF on organ weights and immune function reflected their different modes of action. Cu_Gly increased relative liver weight, possibly due to copper accumulation and metabolic stress, while KDF had no such effect. Both treatments reduced relative spleen weight, which may indicate reduced immune activation due to pathogen suppression [31]. The present study observed that the KDF treatment upregulated the plasma content of IgG and downregulated the plasma contents of IgM and IL-6 in comparison to both the control and Cu_Gly treatment. Additionally, it decreased the plasma content of IL-1 compared to the control piglets. These findings were in line with previous research, which has shown that dietary supplementation of organic acids can increase serum IgG concentration [32] and decrease serum IL-1, IL-6, and TNF-α levels [33]. IgG, as a secondary immune response antibody, and IL-6, a key pro-inflammatory cytokine, are critical markers of immune status [34,35], indicating the superior immunomodulatory effects of KDF. These results were critical for reducing morbidity in commercial settings. Therefore, KDF presented a viable, practical alternative to copper additives by offering a combination of improved feed efficiency, enhanced resilience, and a superior safety profile, even in the absence of a dramatic growth performance boost.

Previous studies have confirmed copper and organic acidifiers as effective antibiotic alternatives [3,6,7]. Consistent with this, both Cu_Gly and KDF reduced pathogenic genera (*Staphylococcus*, *Escherichia-Shigella*, *Brevibacterium*) in the ileum and cecum. These pathogenic bacteria can cause significant disease burden by counteracting host defenses and producing virulence factors to survive the immune responses evoked by infection [36]. In addition, the abundance of ileum *Escherichia-Shigella* and *Staphylococcus* genera negatively correlated with IgG, and the cecum *Escherichia-Shigella* genus positively correlated with IL-6. These results further supported their immunosuppressive effects. Notably, KDF enriched beneficial *Lachnospiraceae_UCG-007* in the cecum, which was negatively correlated with IL-1, similar to the anti-inflammatory effects of other *Lachnospiraceae* taxa reported by Hu et al. (2022) [33]. In contrast, the control group’s enriched *Lachnospiraceae_XPB1014_group* was positively correlated with IL-6, indicating functional diversity within the *Lachnospiraceae* family. In addition, compared to the Control and Cu_Gly groups, the KDF group had *TM7* (*Saccharimonadales*) enrichment in the ileum microbiota. Li et al. (2022) linked *Saccharimonadales* enrichment to reduced fecal inflammatory markers in selenium-supplemented pigs [37], but no direct correlation with plasma cytokines was observed here. Therefore, the role of the *TM7* genus in the gut microbiome should be elucidated in future studies.

A noteworthy observation with potential long-term implications was the enrichment of specific bacterial genera in the Cu_Gly group. This treatment led to increased abundances of *Terrisporobacter* and *Longispora*, which have been linked to heavy metal and antibiotic resistance. Zhang et al. (2022) observed that *Terrisporobacter* was enriched in swine manure containing a higher concentration of copper [38]. Furthermore, the abundance of the heavy metal resistance genes positively correlated with the antibiotic resistance genes in the manure bacterial communities. Sardar et al. (2021) observed that antibiotic treatment increased the abundance of *Longispora* genera with antibiotic-resistant genes in the swine manure [39]. Moreover, Zhang et al. (2015) isolated copper-resistant *Enterococcus faecalis* from copper-fed pigs [40], highlighting the risk of antibiotic resistance gene dissemination. While the present study did not directly quantify copper or antibiotic resistance genes, the enrichment of bacterial taxa historically linked to heavy metal resistance in the Cu_Gly group suggests a potential risk for promoting resistant populations. This contrasts with the mode of action of KDF, which avoids metal supplementation. The enrichment of such taxa underlines a potential drawback of copper-based additives that is absent with KDF, reinforcing KDF’s position as a more sustainable alternative from a microbial ecology and antimicrobial resistance perspective.

## 5. Conclusions

Dietary supplementation with 10 g/kg KDF or 60 mg/kg Cu_Gly effectively suppressed gut pathogenic bacteria in weaned piglets fed copper-unsupplemented diets. However, Cu_Gly may increase the risk of copper-resistant bacteria. KDF exhibited superior immunomodulatory effects by enhancing plasma IgG levels and reducing pro-inflammatory cytokines (IL-1, IL-6), which was associated with the enrichment of beneficial taxa (*Lachnospiraceae_UCG-007*, *TM7*). These findings demonstrated that KDF represented a viable and sustainable alternative to copper supplements, offering a combination of improved gut health, enhanced immunity, and a reduced environmental risk profile.

## Figures and Tables

**Table 1:** Compositions and nutrient levels in basal diets (as-fed basis).

Diet	Content (%)
Ingredients	
Corn	70.00
Soybean meal	24.00
Premix ^1^	6.00
Total	100.00
Nutrient levels	
Digestive energy, Mcal·kg^−1^	3.13
Crude protein, %	16.09
Lysine, %	1.40
Methionine + cystine, %	0.84
Threonine	0.95
Tryptophan	0.31
Calcium, %	0.74
Phosphorus, %	0.60

^1^ The premix provides following for per kg diets: Phytase 500 IU, Zn 60 mg, Fe 150.3 mg, Mn 85.9 mg, Se 0.3 mg, I 0.14 mg, vitamin A 2000 IU, vitamin D3 1500 IU, vitamin E 53 mg, vitamin K3 1 mg, vitamin B1 6 mg, vitamin B2 2.8 mg, vitamin B3 8 mg, vitamin B5 28 mg, vitamin B6 2.8 mg, vitamin B9 2 mg, vitamin B12 0.1 mg, Biotin 0.2 mg, lysine 7.6 g, methionine 3.4 g, Threonine 3.4 g, Tryptophan 1.3 g, Isoleucine 1.8 g, Valine 1.9 g, CaCO_3_ 10.3 g, CaHPO_3_ 13 g, NaCl 3.5 g, Choline chloride 1.2 g.

**Table 2:** Growth performance and relative organ weight ^1^.

Items	Control	Cu_Gly	KDF	*p* Value		
				Cu_Gly vs. Control	KDF vs. Control	KDF vs. Cu_Gly
Initial BW, kg	8.14 ± 0.49	8.64 ± 0.38	8.92 ± 0.41	0.425	0.221	0.625
Final BW, kg	19.29 ± 0.93	20.80 ± 0.66	20.27 ± 0.93	0.231	0.429	0.666
ADG, g/d	227.47 ± 48.74	248.08 ± 48.98	231.55 ± 45.97	0.570	0.408	0.505
ADFI, g/d	528.57 ± 86.61	555.18 ± 108.09	505.63 ± 86.61	0.138	0.489	0.309
FCR (feed/gain)	2.44 ± 0.21 ^b^	2.34 ± 0.32 ^ab^	2.27 ± 0.18 ^a^	0.015	0.609	0.758
Relative organ weight, g/kg of BW						
Liver	22.78 ± 0.44 ^b^	24.67 ± 0.49 ^a^	23.75 ± 0.63 ^ab^	0.026	0.218	0.241
Spleen	1.60 ± 0.09 ^a^	1.13 ± 0.10 ^b^	1.14 ± 0.09 ^b^	0.006	0.007	0.918

^1^ Control, basal diet. Cu_Gly, basal diet + 60 mg/kg Cu-Glycine. KDF, basal diet + 10 g/kg potassium diformate. Data are represented as means ± SEM. In the same row, data with the same letter superscripts mean a significant difference between different groups, *p* < 0.05, n = 5. BW, body weight; ADG, average daily gain; ADFI, average daily feed intake; FCR, feed conversion ratio.

**Table 3:** Plasma contents of immunoglobulin and cytokines ^1^.

Items	Control	Cu_Gly	KDF	*p* Value		
				Cu_Gly vs. Control	KDF vs. Control	KDF vs. Cu_Gly
IgA, mg/mL	2.06 ± 1.00	1.89 ± 0.30	2.06 ± 1.00	0.716	0.506	0.745
IgG, mg/mL	31.80 ± 3.07 ^b^	30.59 ± 2.98 ^b^	31.80 ± 3.07 ^a^	0.745	0.010	0.005
IgM, mg/mL	17.92 ± 1.45 ^a^	15.95 ± 2.12 ^a^	17.92 ± 1.45 ^b^	0.412	0.003	0.016
IL-1, pg/mL	20.78 ± 1.48 ^a^	17.44 ± 1.96 ^ab^	20.78 ± 1.48 ^b^	0.195	0.050	0.433
IL-2, pg/mL	515.84 ± 39.33	465.84 ± 32.93	515.84 ± 39.33	0.425	0.305	0.810
IL-4, pg/mL	130.71 ± 21.31	129.41 ± 22.26	130.71 ± 21.31	0.966	0.894	0.927
IL-6, pg/mL	354.68 ± 29.59 ^a^	348.72 ± 52.22 ^a^	354.68 ± 29.59 ^b^	0.910	0.037	0.046
IL-10, pg/mL	547.42 ± 39.93	544.32 ± 45.97	547.42 ± 39.93	0.958	0.915	0.874
TNF-α, pg/mL	102.59 ± 8.04	99.44 ± 4.39	102.59 ± 8.04	0.701	0.621	0.911
IFN-γ, pg/mL	102.72 ± 1.57	104.05 ± 3.96	102.72 ± 1.57	0.737	0.980	0.755

^1^ Control, basal diet. Cu_Gly, basal diet + 60 mg/kg Cu-Glycine. KDF, basal diet + 10 g/kg potassium diformate. Data are represented as means ± SEM. In the same row, data without the same letter superscripts mean a significant difference between different groups, *p* < 0.05, n = 5.

**Table 4:** Alpha diversity indices of ileum and cecum bacteria ^1^.

Items	Control	Cu_Gly	KDF	*p* Value		
				Cu_Gly vs. Control	KDF vs. Control	KDF vs. Cu_Gly
Ileum						
OTUs	359.80 ± 22.45 ^b^	545.80 ± 45.73 ^a^	464.80 ± 69.13 ^ab^	0.021	0.161	0.272
Ace	629.76 ± 52.69	753.64 ± 52.22	672.04 ± 46.73	0.109	0.556	0.277
Chao	531.13 ± 39.23 ^b^	702.77 ± 38.76 ^a^	619.53 ± 73.44 ^ab^	0.041	0.261	0.289
Shannon	2.40 ± 0.26 ^b^	3.26 ± 0.24 ^a^	3.20 ± 0.30 ^ab^	0.042	0.056	0.875
Simpson	0.29 ± 0.08 ^a^	0.10 ± 0.02 ^b^	0.13 ± 0.05 ^ab^	0.026	0.054	0.694
Coverage, %	99.66 ± 0.00	99.58 ± 0.00	99.60 ± 0.00	0.142	0.215	0.799
Cecum						
OTUs	850.00 ± 46.36	833.40 ± 54.09	737.40 ± 43.70	0.812	0.125	0.185
Ace	962.90 ± 47.07	977.04 ± 60.52	898.73 ± 46.02	0.850	0.397	0.305
Chao	976.70 ± 41.99	985.61 ± 58.20	906.14 ± 31.95	0.892	0.293	0.239
Shannon	4.62 ± 0.09	4.55 ± 0.14	4.43 ± 0.16	0.699	0.333	0.351
Simpson	0.05 ± 0.01	0.05 ± 0.01	0.05 ± 0.01	0.900	0.779	0.686
Coverage, %	99.43 ± 0.04	99.37 ± 0.05	99.43 ± 0.02	0.278	0.981	0.268

^1^ Control, basal diet. Cu_Gly, basal diet + 60 mg/kg Cu-Glycine. KDF, basal diet + 10 g/kg potassium diformate. The operational taxonomic units (OTUs) were defined with 97% similarity. The coverage percentages, richness estimators (Ace and Chao), and diversity indices. The data were expressed as mean value ± SEM, n = 5. In the same row, data with the same letter superscripts mean a significant difference between different groups, *p* < 0.05.

## Data Availability

The data supporting the findings of this study are available within the article/Supplementary Material; there is no undisclosed data in this study.

## Supplementary Materials

The following supporting information can be downloaded at: https://www.mdpi.com/article/10.3390/ani16121889/s1, Table S1: Pearson correlation analysis between the ileum bacteria and the plasma content of Immune globulin and cytokines; Table S2: Pearson correlation analysis between the cecum bacteria and the plasma content of Immune globulin and cytokines.

## Abbreviations

The following abbreviations are used in this manuscript:

ICu_Gly	copper glycinate
KDF	potassium diformate
ADG	average daily body weight gain
ADFI	average daily feed intake
OTU	operational taxonomic units
LEfSe	linear discriminant analysis effect size
