# Supplementary material for: Comparative Effects of Copper Glycinate and Potassium Diformate on Immunity and Gut Microbiota of Pigs—Potential Analysis of Potassium Diformate as a Copper Additive Substitute
Source: Animals (Basel). 2026 Jun 18;16(12):1889. doi: 10.3390/ani16121889 (PMC13296155; doi:10.3390/ani16121889)
Supplement: Supplementary file 1 [file animals-16-01889-s001.zip › animals-4340537-supplementary.pdf]

Supplementary Materials

Table S1 Pearson correlation analysis between the ileum bacteria and the plasma content of Immune globulin and cytokines [1]

Biomarked bacteria	IgG	IgM	IL-1	IL-6
Staphylococcus	-0.55 *	0.58 *	0.42	0.51
Escherichia-Shigella	-0.76 **	0.56 *	0.50	0.65 **
Brevibacterium	-0.29	0.41	0.33	0.50
Terrisporobacter	-0.19	-0.10	-0.28	0.06
norank_f_norank_o_Actinomarinales	0.13	-0.24	-0.49	-0.21
norank_f_norank_o_SBR1031	0.16	-0.04	-0.25	-0.08
Bacillus	-0.21	-0.22	-0.45	-0.16
Longispora	0.31	-0.21	-0.29	-0.41
S0134_terrestrial_group	0.00	-0.18	-0.35	-0.15
TM7	0.37	-0.33	-0.49	-0.47
Nesterenkonia	0.51	-0.47	-0.49	-0.39

[1] Only significant enriched bacteria and significant differential immune globulin and cytokines were analysised. Control, basal diet. Cu_Gly, basal diet + 60 mg/kg Cu-Glycine. KDF, basal diet + 10g/kg potassium diformate. N = 5. * means $P < 0.05$, ** indicates $P < 0.01$.

Table S2 Pearson correlation analysis between the cecum bacteria and the plasma content of Immune globulin and cytokines [1]

Biomarked bacteria	IgG	IgM	IL-1	IL-6
Lachnospiraceae_XPB1014_group	-0.48	0.46	0.51	0.54 *
Escherichia-Shigella	-0.52 *	0.35	0.37	0.56 *
norank_f_UCG-010	-0.26	0.32	0.432	0.34
Lachnospiraceae_UCG-007	0.16	-0.21	-0.60 *	-0.45

[1] Only significant enriched bacteria and significant differential immune globulin and cytokines were analysised. Control, basal diet. Cu_Gly, basal diet + 60 mg/kg Cu-Glycine. KDF, basal diet + 10g/kg potassium diformate. N = 5. * means $P < 0.05$, ** indicates $P < 0.01$.
